# Social medias in increasing anxiety around COVID-19 in Morocco

**DOI:** 10.1192/j.eurpsy.2021.1774

**Published:** 2021-08-13

**Authors:** I. Hanine, M. Chtibi, Y. Bensalah, S. Belbachir, A. Ouanass

**Affiliations:** 1 Psychiatrie, Hôpital Ar-razi de Salé, Rabat, Morocco; 2 Psychiatry, Hopital Arrazi e Salé, Maroc, Rabat, Morocco

**Keywords:** COVID-19, Anxiety, well-being, who-5

## Abstract

**Introduction:**

Discovered in December 2019, COVID has affected the entire planet, through direct exposure to its virus; SARS-COV-2, or indirectly through the media, Indeed, on January 20, 2020, the World Health Organization declared COVID-19 to be “a public health emergency of international concern.” Along with other public health crises and other collective trauma (terrorism, H1N1 epidemic or SARS-COV), exposure to publicized information on this virus generates psychiatric disorders, in particular anxiety and absence of well-being. Objective: To link exposure to information about this pandemic through social media and anxiety and lack of well-being.

**Objectives:**

Explore the relationship between anxiety, well-being and exposure to social medias

**Methods:**

Use of a questionnaire consisting of three sections, individual status and conditions, the French versions of the GAD-7 scale for anxiety (Generalized anxiety scale of 7items) and the WHO-5 (five well-being index). This questionnaire is dedicated to the general population who have not been in direct contact with the virus, but through the media.

**Results:**

We were able to collect 209 participants, they were essentially females with a mean age of 28yo, 17,7% had psychiatric history of anxiety and depression, the median use of social medias was 5.7 hours per day. And they were essentially getting their information about the pandemic from Instagram, Facebook, the Moroccan ministry of health’s website and electronic newspapers. 31,1% of our participants had anxiety which was above a Chinese study, and had a poor well-being.

**Conclusions:**

The use of social media to get information about the pandemic had an impact on well-being and anxiety.

**Disclosure:**

No significant relationships.

